# The Rare Benign Lesion That Mimics a Malignant Tumor in Breast Parenchyma: Nodular Fasciitis of the Breast

**DOI:** 10.1155/2018/1612587

**Published:** 2018-04-30

**Authors:** Hilal Erinanc, Emin Türk

**Affiliations:** ^1^Medicine Faculty, Pathology Department, Konya Uygulama ve Arastırma Hastanesi, Baskent University, Selcuklu, Konya, Turkey; ^2^Medicine Faculty, Surgery Department, Konya Uygulama ve Arastırma Hastanesi, Baskent University, Selcuklu, Konya, Turkey

## Abstract

We herein report the clinical and pathological findings of a rare case of nodular fasciitis in the breast parenchyma of a 48-year-old female. Because of potentially malignant findings on ultrasonography and during clinical examination, the patient underwent an excisional biopsy. Histologically, the lesion was composed of spindle to round shaped cells arranged in short bundles in a storiform pattern. Immunohistochemically, the cells were positive for vimentin and SMA and negative for desmin, S100, and CD34. Based on these morphological and immunohistochemical features, a diagnosis of nodular fasciitis was made. We emphasize that nodular fasciitis of the breast may show clinical features and imaging findings similar to those of breast cancer. The histopathologic diagnosis of nodular fasciitis can also be challenging. The purpose of this case report is to highlight the characteristics and the differential diagnosis of this rare neoplasm.

## 1. Introduction

Nodular fasciitis is a benign proliferative lesion of soft tissue with unknown etiology. Although extremely rare in the breast, nodular fasciitis may occur anywhere in the body and can involve different organs. The clinical presentation is characterized by a rapidly growing mass that may be painful or tender. It may lead to differential diagnostic problems because it may clinically, radiographically, and histologically mimic a malignant tumor. Histologically, the major differential diagnostic considerations are malignant spindle cell tumors and fibromatosis [1].

In this article, we report a case of nodular fasciitis of the breast, with emphasis on the histological characteristics of these lesions, and discuss the differential diagnosis.

## 2. Case Presentation

**Figure 1:**
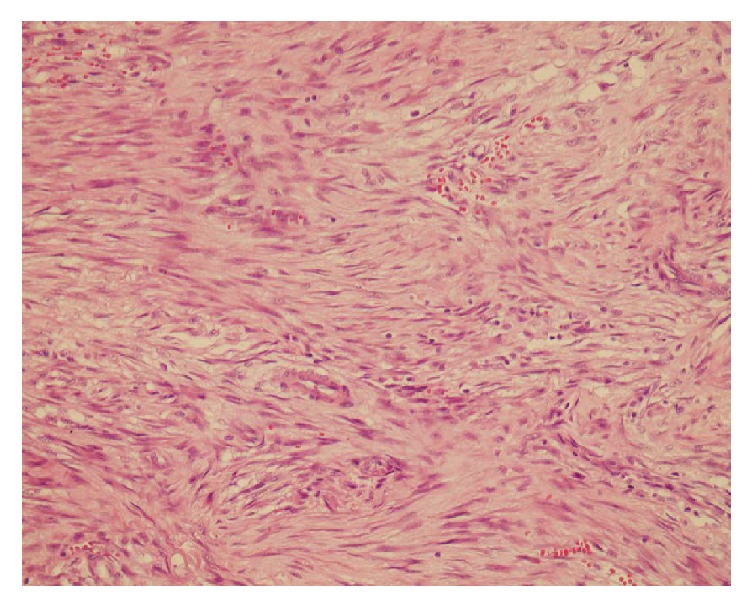
Picture shows that spindle cell proliferation admixed with inflammatory cells (HE ×40).

**Figure 2:**
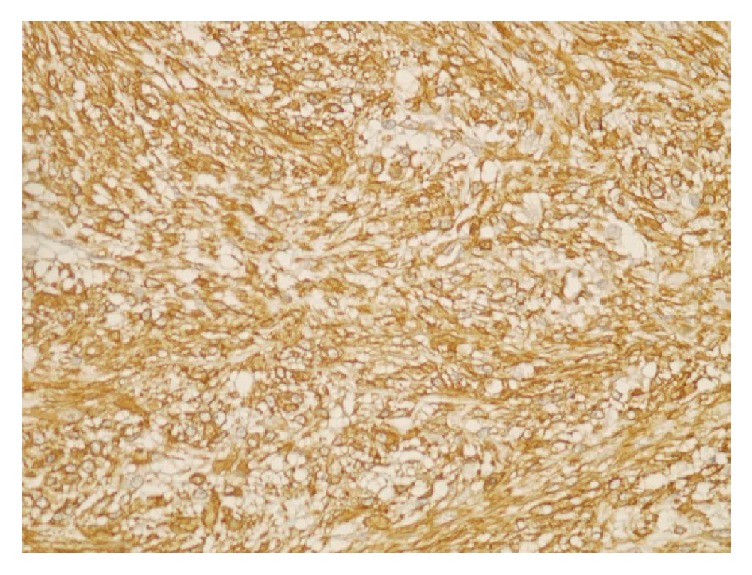
Positive cytoplasmic staining with SMA in nodular fasciitis (SMA ×40).

The 48-year-old woman was admitted to our hospital with complaints of mild pain and a palpable mass in her left breast. There was no family history of breast cancer. On examination, there was a small nodule located underneath the areola and measuring about 2 cm in maximum diameter. Ultrasonography revealed a heterogenic hypoechoic lesion with infiltrative margins in the breast parenchyma, measuring 13 × 9 mm, which had millimetric calcification foci and extended to the subcutaneous tissue. The excised mass consisted of multiple fragments of irregular and white soft tissue, measuring about 2.5 × 2 × 2 cm. Sectioning revealed a whitish, fibrous, and fatty lobular cut surface with no gross distinguishing marks. Microscopy showed the tumor was composed of spindle and mildly polygonal cells arranged in short bundles (Figure 1). The tumor also had an irregular infiltrative margin that invaded into the adipose tissue. Numerous normal mitotic figures were present. There was no breast tissue in the tumor. Foci of myxoid degeneration, inflammation, and occasional multinucleated cells were found, concordant with the histologic pattern of nodular fasciitis. The tumor margins could not be evaluated due to the fragmentized nature of the specimen. Immunohistochemical examination showed that the tumor cells stained for smooth muscle actin (SMA) and vimentin while they did not stain for desmin, S100, and CD34 (Figure 2).

## 3. Discussion

Nodular fasciitis of the breast is a rare and reactive process composed of fibroblasts and myofibroblasts. Breast tumor classification has been revised, and nodular fasciitis of the breast was added to the World Health Organization classification in 2012 as one of the benign mesenchymal breast tumors [2].

Clinically, most patients have a history of a rapidly growing mass or nodule that has been present for only 1-2 weeks [3]. Nodular fasciitis is most common in adults between 20 and 40 years of age, and it usually arises in the subcutaneous tissue or less often in the mammary parenchyma and occurs as a solitary lesion that is usually less than 3 cm in diameter. While it is believed that local injury may play a role in the fibroblastic proliferation, one study showed a history of trauma was described in only 10% of patients [4]. There was also no history of trauma in our case. Because of its infiltrating margins, mammography and ultrasound findings may also suggest malignancy [5]. Authors have reported that the appearance of nodular fasciitis in the breast may mimic intraductal carcinoma [6]. Nodular fasciitis is rarely diagnosed by fine needle aspiration cytology [7] or core needle biopsy, and it usually requires excisional biopsy for histologic confirmation. While authors have reported the proliferation of neoplastic spindle cells was suspected, no definitive diagnosis was obtained with aspiration cytology [8]. Therefore surgical excision provided to examine whole specimen is important to obtain final diagnosis.

Histologically, it can show a certain degree of cell mitosis and cellularity, which can raise suspicion of malignancy, even though they are benign. Authors have emphasized that the most common diagnostic difficulty arises with other benign and malignant spindle cell tumors (including spindle cell carcinomas and sarcomas) and fibromatosis [1]. In the present case, we initially thought that the lesion resembled leiomyoma. However, the spindle cells showed positive staining for SMA and vimentin and negative staining for desmin. These results suggested a myofibroblastic origin for the tumor cells. Both myofibroblastic and smooth muscle neoplasms can display immunoreactivity for desmin, muscle specific actin, and SMA; however, desmin is expressed more frequently in smooth muscle tumors. In our case, nodular fasciitis was diagnosed based on the immunostaining results (positivity for SMA and vimentin, negativity for S100, CD34, and desmin), in addition to the morphological findings. The literature generally points out that the key features for diagnosis are the presence of inflammatory cells (mainly lymphocyte) and extravasated red blood cells with clusters of reactive fibroblasts that are arranged in short bundles in a prominent myxoid stroma, as in our case [9]. Some cases of nodular fasciitis can be difficult to distinguish from fibromatosis. Histologically, fibromatosis is characterized by slender shaped fibroblasts arranged in long sweeping fascicles in a uniformly collagenous matrix, and they generally lack an inflammatory component. Primary sarcomas of the breast are also considered in differential diagnosis. Distinction from sarcoma is primarily a matter of growth pattern cellularity and mitotic activity. The cells in sarcoma are marked by a greater variation in size and shape, hyperchromatic nuclei, and a more pronounced mitotic rate, including atypical mitotic figures. The absence of these nuclear features may help differentiate nodular fasciitis from sarcomas. In addition some spindle cell metaplastic carcinoma may appear with lack of epithelial differentiation; however they usually show at least reactivity for one or more keratins.

The etiology of nodular fasciitis is uncertain. Although it is considered a reactive proliferation and some patient may have a history of trauma to the site of the lesion, it has been reported that rare cases have clonal chromosomal abnormalities, which may suggest some nodular fasciitis cases to be a clonal myofibroblastic tumor [10].

The treatment for nodular fasciitis is surgical excision, which is curative. Recurrence after surgical excision is rare [11]. Paliogiannis et al. reported that no recurrences were observed in the breast cases when they were reviewed [4]. In addition spontaneous regression is reported in breast and other tissues. Because of the spontaneous regression probability, some authors advise only careful observation [12, 13]. There is also a reported case of biopsy-proven nodular fasciitis, resolving completely after an intralesional corticosteroid injection [14].

In conclusion, the development of nodular fasciitis in the breast can resemble malignant processes. In addition, histologic features, such as cellularity and mitosis, may be misinterpreted as a malignancy. Although nodular fasciitis rarely occurs in the breast, it should be considered in differential diagnosis of spindle cell lesions in the breast to avoid overdiagnosis.
